# Multimodal fluorescence lifetime imaging and optical coherence tomography for longitudinal monitoring of tissue-engineered cartilage maturation in a preclinical implantation model

**DOI:** 10.1117/1.JBO.28.2.026003

**Published:** 2023-02-16

**Authors:** Xiangnan Zhou, Anne K. Haudenschild, Cai Li, Laura Marcu

**Affiliations:** University of California Davis, Department of Biomedical Engineering, Davis, California, United States

**Keywords:** engineered tissues, fluorescence lifetime, optical coherence tomography, tissue optical spectroscopy and imaging, cartilage

## Abstract

**Significance:**

Cartilage tissue engineering is a promising strategy for effective curative therapies for treatment of osteoarthritis. However, tissue engineers depend predominantly on time-consuming, expensive, and destructive techniques as quality control to monitor the maturation of engineered cartilage. This practice can be impractical for large-scale biomanufacturing and prevents spatial and temporal monitoring of tissue growth, which is critical for the fabrication of clinically relevant-sized cartilage constructs. Nondestructive multimodal imaging techniques combining fluorescence lifetime imaging (FLIm) and optical coherence tomography (OCT) hold great potential to address this challenge.

**Aim:**

The feasibility of using multimodal FLIm–OCT for nondestructive, spatial, and temporal monitoring of self-assembled cartilage tissue maturation in a preclinical mouse model is investigated.

**Approach:**

Self-assembled cartilage constructs were developed for 4 weeks *in vitro* followed by 4 weeks of *in vivo* maturation in nude mice. Sterile and nondestructive *in situ* multispectral FLIm and OCT imaging were carried out at multiple time points (t=2, 4, and 8 weeks) during tissue development. FLIm and 3D volumetric OCT images were reconstructed and used for the analysis of tissue biochemical homogeneity, morphology, and structural integrity. A biochemical homogeneity index was computed to characterize nonhomogeneous tissue growth at different time points. OCT images were validated against histology.

**Results:**

FLIm detects heterogenous extracellular matrix (ECM) growth of tissue-engineered cartilage. The outer edge of the tissue construct exhibited longer fluorescence lifetime in 375 to 410 and 450 to 485 nm spectral channels, indicating increase in collagen content. Significant (p<0.05) decrease of construct homogeneity index was observed between t=2 weeks and t=4 weeks. Both FLIm and OCT images revealed defects (voids) at the center of the tissue construct during *in vitro* culture (t=2 and 4 weeks). Cyst formation during *in vivo* culture was detected by OCT and confirmed with histology.

**Conclusions:**

The ability of multimodal FLIm–OCT to nondestructively monitor the heterogenous growth of engineered tissue constructs *in situ* is demonstrated. Spatial and temporal variation of construct ECM component was detected by FLIm. OCT reveals structural defects (voids and cysts). This multimodal approach has great potential to replace costly destructive tests in the manufacturing of tissue-engineered medical products, facilitating their clinical translation.

## 1 Introduction

Damage to articular cartilage, which can occur through traumatic injury, pathology, or age, often degenerates inexorably to osteoarthritis (OA). Unfortunately, adult articular cartilage has only limited ability to heal as cartilage tissue is avascular and hypocellular.^1^^,^^2^ A multitude of treatments have been developed for this age-long problem, but no satisfactory long-term therapies are established. In the United States alone, OA affects the quality of lifetime for more than 20 million Americans and cost the economy exceeding $65 billion per year (both medical costs and lost wages).^3^

Cartilage tissue engineering is an emerging strategy at the threshold of clinical translation. It holds immense potential to improve the clinical outcome and deliver effective curative therapies for treatment of OA.^4^ Currently, in both research and clinical setting, tissue engineers depend predominantly on a battery of time-consuming, expensive, and destructive techniques as quality control to monitor the composition, structure, and function of engineered tissue during culture.^5^ Destructive testing can be inappropriate or impractical for large-scale biomanufacturing as partial or complete sample loss is required. It has been estimated that 70% of the cost of tissue engineered goods can be attributed to quality control efforts.^6^

Furthermore, destructive testing often prevents spatial or temporal monitoring during tissue maturation which are critical for fabrication of clinical-relevant-sized tissue construct. To date, small cartilage tissue constructs (∼Ø4×2  mm) with biochemical content and mechanical function approaching native cartilage have been successfully fabricated.^7^ However, a major challenge remains in the development of clinically relevant-sized tissue constructs, typically 15 to 25 mm in diameter and as thick as 5 mm, which are required to repair OA defects.^8^ Larger engineered cartilage constructs often suffer from highly heterogeneous matric deposition and possess poor mechanical properties that are unable to support physiologic loading.^9^^–^^11^ Therefore, there is an immediate need to develop nondestructive and noninvasive tools to not only verify the critical biochemical and structural component but also monitor the spatial and temporal growth of tissue engineered cartilage products for both research and clinical applications.

Optical techniques have shown great potential to address this unmet need due to their capacity to probe tissue composition and structure spatially and temporally in a nondestructive and noninvasive manner. Fluorescence lifetime-based techniques are known to detect biochemical and functional alternations in tissue.^12^^,^^13^ Several endogenous fluorophores, including the extracellular matrix (ECM) structural proteins (e.g., collagen) and enzyme cofactors [e.g., nicotinamide adenine dinucleotide (NADH) and flavin adenine dinucleotide (FAD)], are responsible for tissue autofluorescence.^14^ Fluorescence lifetime imaging (FLIm) based on autofluorescence provides a sensitive method for label-free nondestructive monitoring of bioengineered tissues both *in vitro* and *in vivo*. The spatial distribution of tissue biochemical component can be mapped by FLIm, providing critical information to assess the heterogeneous growth of large engineered construct. Recently, FLIm have been applied for the characterization of engineered vascular construct,^15^ osteogenic grafts,^16^^,^^17^ and cartilage construct.^18^^,^^19^

Optical coherence tomography (OCT), which is based on low-coherence interferometry, is an imaging modality analogous to ultrasound imaging (US) but uses near-infrared radiation rather than sound waves.^20^ OCT is well recognized as a viable tool for studying the structure and morphology of biological tissue for diagnosis of diseases,^21^ such as atherosclerosis,^22^ epithelial cancer,^23^ OA,^24^ and for surgical guidance.^25^ State-of-the-art OCT systems can image cartilage tissue at the micron resolution (near-histological) and 1 to 2 mm in depth.^24^ Real-time 2D cross-sectional or 3D volumetric scans are easily achievable. Compared with US, which has been used to evaluate engineered tissue constructs,^19^^,^^26^^,^^27^ OCT offers better resolution, higher imaging speed, and easy integration with other optical imaging modalities. Recently, OCT has been explored as a promising tool for real-time monitoring of the development and growth of tissue-engineered products.^28^

The monitoring of tissue engineered cartilage could benefit from the bimodal diagnostic approach combining FLIm and OCT techniques. FLIm can investigate construct biochemical composition but is limited to the thin layers (<0.5  mm) within the penetration depth of UV light. However, little structure information of the tissue construct is retrieved. In contrast, OCT enables three-dimensional evaluation of tissue microstructure, morphology, and potential structural defects, but lacks information on tissue biochemical composition. Thus FLIm and OCT can complement each other and together provide a more comprehensive orthogonal noninvasive spatial characterization and evaluation of cartilage constructs than either modality alone. Moreover, the two modalities can be easily integrated using an optical fiber probe, enabling sterile, longitudinal assessment of engineered tissue.^29^ The ability of FLIm-OCT to probe biochemical and structural component of tissue spatially and nondestructively makes it a promising tool for characterization of engineered cartilage tissue.

The overall objective of this study was to evaluate the ability of a bimodal system combing FLIm and OCT for nondestructive, spatial, and longitudinal monitoring of changes in biochemical and structural properties of self-assembled cartilage construct in a preclinical implantation model. Chondrogenesis has been extensively studied in murine models, in which self-assembled cartilage constructs are implanted in dorsal subcutaneous pouches of immunocompromised nude mice and this model is the first step in evaluating the chondrogenic potential of tissue-engineered cartilage *in vivo*.^30^ A fiber-based multimodal FLIm-OCT system was used to realize label-free monitoring of key biochemical and structural markers at three key time points (t=2, 4, and 8 weeks) during engineered tissue maturation without perturbing or compromising sample sterility. Specifically, the goals of this study were to determine whether bimodal FLIm-OCT can *in situ* assess (1) the heterogeneous construct growth in an *in vitro* self-assembled construct culture model and (2) tissue maturation in an *in vivo* preclinical animal model.

## 2 Materials and Methods

### 2.1 Chondrocyte Isolation

Articular cartilage was harvested from the femoral condyles of five juvenile bovine stifle joints (Research 87, Boston, Massachusetts, USA), minced, and digested in Dulbecco’s Modified Eagle Medium (DMEM) with high glucose/GlutaMAX™-I (Life Technologies, Grand Island, New York, USA) with 0.3% collagenase type II (Worthington, Lakewood, New Jersey, USA), 5% fetal bovine serum (HyClone, GE Healthcare Life Sciences, Marlborough, Massachusetts, USA), and 1% penicillin/streptomycin/fungizone (P/S/F) (Lonza, Basel, Switzerland) for 18 h on an orbital shaker at 37°C. Following digestion, the cells were collected, pooled, filtered through 70-μm cell strainers, and washed three times with DMEM.

### 2.2 In Vitro Self-Assembled Construct Culture

**Fig. 1:**
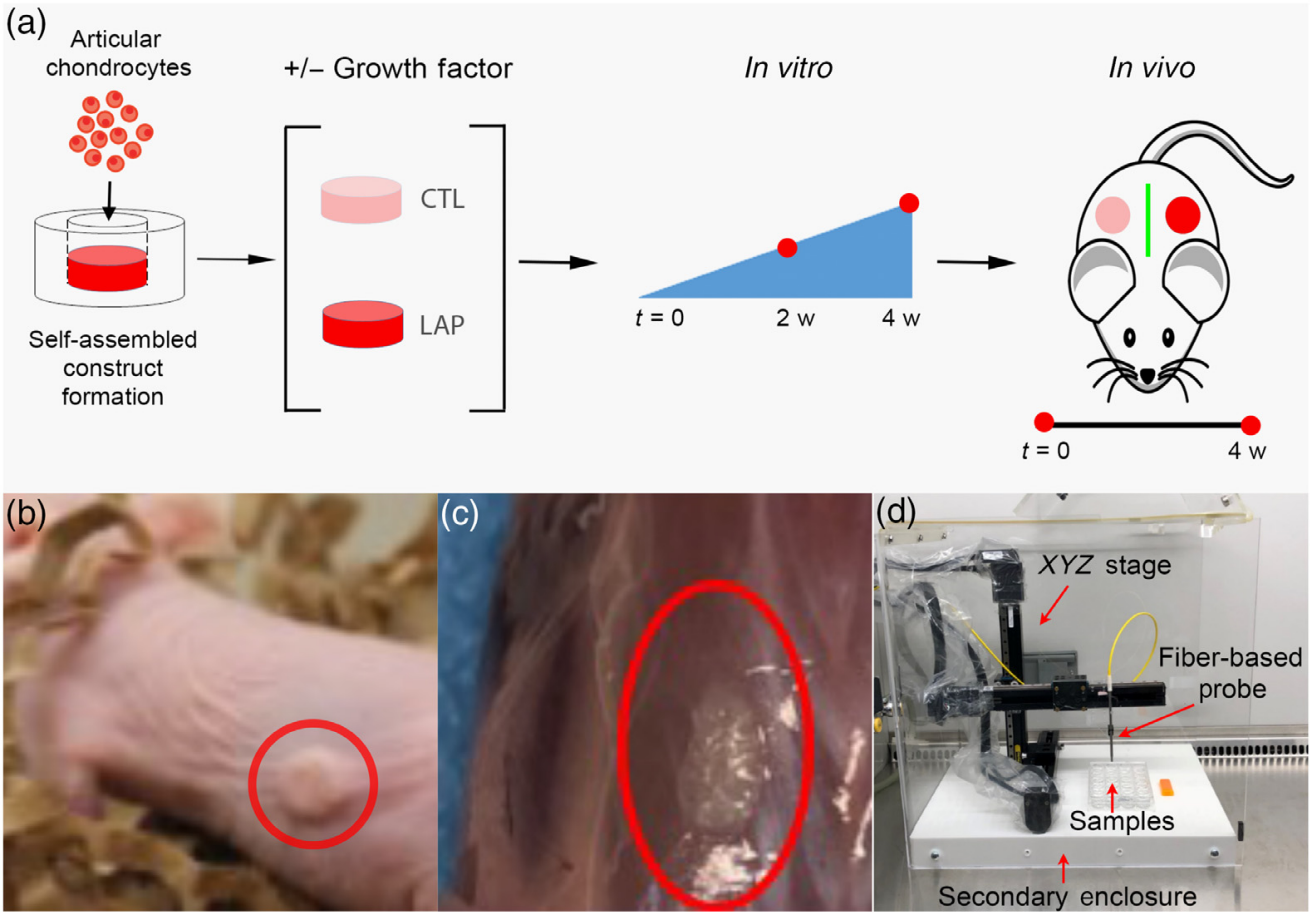
(a) Schematic overview of the experiment design. Self-assembled constructs were treated with different growth factor and cultured *in vitro* for 4 weeks, followed by *in vivo* maturation of another 4 weeks. Multimodal FLIm-OCT imaging was carried out at t=2 weeks and 4 weeks (0 week *in vivo*) during *in vitro* culture and at t=4 weeks for *in vivo* culture. Red dots indicate imaging time points. (b) Tissue construct following implantation on the back of nude rat. (c) Engineered tissue after 4 weeks of *in vivo* culture following sacrifice of the animal. (d) Picture of the scanning setup inside biosafety cabinet.

Engineered cartilage constructs were formed using the self-assembling process, as described previously^31^ [Fig. 1(a)]. Briefly, $4\times 10^{6}$ chondrocytes were suspended in 100  μL of control medium (CTL) consisting of DMEM with 1% ITS+ Premix (BD Biosciences, Bedford, Massachusetts, USA), 1% nonessential amino acids (Life Technologies), 50  μg/mL ascorbate-2-phosphate (Sigma-Aldrich, St. Louis, Missouri, USA), 40  μg/mL L-proline (Sigma-Aldrich), 100  μg/mL sodium pyruvate (Sigma-Aldrich), 100 nM dexamethasone (Sigma-Aldrich), and 1% P/S/F. Cell suspensions were seeded in 5 mm diameter, 2% agarose wells in 24-well plates (Costar, Corning, New York, USA). After 4 h, 400  μL of control medium were added to each well. Self-assembled constructs were cultured for 4 weeks in (1) CTL medium only (CTL) (five samples) or (2) with recombinant human LAP transforming growth factor beta (TGF-β1) (LAP) (five samples) (R&D Systems, Minneapolis, Minnesota)^32^ applied at 10  ng/mL for the entire culture duration. Constructs were removed from wells at day 5, and 1 mL of fresh media was exchanged daily.

### 2.3 In Vivo Self-Assembled Construct Culture

Following 4 weeks of *in vitro* culture, constructs were implanted subcutaneously into the dorsal section of 5 nude mice [Fig. 1(b)], in which the left side was the LAP group, and the right side was the CTL group. After 4 weeks of *in vivo* culture, the mice were sacrificed, and all surviving samples were harvested and processed for analysis [Fig. 1(c)].

### 2.4 Multimodal Assessment of Self-Assembled Articular Cartilage

A multimode imaging system that combines two complementary techniques of FLIm and OCT was utilized to make nondestructive assessment of the engineered cartilage construct at 2 weeks and 4 weeks of *in vitro* culture (n=5 per group) and 4 weeks of *in vivo* maturation (n=4 per group) [Fig. 1(d)]. Samples were imaged using FLIm (resolution of 100  μm) followed immediately by OCT in a sterile environment inside a biosafety cabinet. Matched samples were assessed nondestructively (FLIm–OCT) at all time point and histological analysis was performed at 4 weeks *in vivo*.

### 2.5 Multispectral FLIm Instrument

The multispectral FLIm subsystem has been reported in detail in previous publications.^33^ Briefly, a Q-switched pulsed 355-nm microchip laser (STV-02E-1x0, TEEM photonics, Grenoble, France) with 2-μJ pulse energy and 600-ps pulse duration was used to excite sample autofluorescence through a flexible fiber-optic probe consist of 400  μm core diameter pure silica multimode fiber (FVP400440480, Polymicro Optical Fiber, Molex, Illinois, USA). Sterile imaging was achieved by raster scanning the fiber probe across the surface of sample using a three-axis translation stage (LP28, Parker, Cleveland, Ohio, USA) housed inside a biosafety cabinet. During scanning, each sample was placed inside a sterile 35 mm glass bottom dish (MatTech Corporation, Ashland, Massachusetts, USA) in phosphate buffered saline at room temperature. Sample autofluorescence emission was separated into four distinct spectral channels (CH1=375 to 410 nm, CH2=450 to 485 nm, CH3=532 to 565 nm, and CH4=595 to 660 nm) using a custom-built wavelength selection module, time-multiplexed using optical fibers of different lengths, detected by a single microchannel plate photomultiplier tube and digitized by a high-speed digitizer (PXIe-5185, National Instrument, Austin, Texas, USA) with a time resolution of 80 ps. Complete mapping of sample surface was achieved under 1 min at a resolution of 100  μm resolution.

### 2.6 FLIm Parameters

Following the acquisition of the fluorescence decay signal, nonnegative constrained least-square deconvolution based on Laguerre expansion method was used to recover the fluorescence response of the tissue sample.^34^ The average lifetime and intensity ratio were derived from the deconvolved fluorescence decay. The average lifetime was defined as the average amount of time a fluorophore stayed in the excited state. Mathematically, it is the expected value of the probability distribution of detected photons, which was obtained by normalizing the deconvolved decay by the total area under the curve. Intensity ratios were defined as the ratio of fluorescence intensity at each channel to the sum of all four intensity channels.

### 2.7 OCT Instrument

The OCT subsystem was a swept source OCT developed based on an OEM OCT engine from Axsun Technology.^35^ Briefly, it includes a 1310±55  nm swept-source laser with 50 mW output power and a native A scan rate of 50 kHz. The OCT interferometry is realized with fiber couplers and circulators that split the laser output into sample and reference arm and recombine the reflected light from both arms. A single-mode fiber (SMF-28 Ultra) is used in the reference arm to match the optical path length introduced by optical fiber probe consist of the same single-mode fiber with a graded-index lens (1.8-mm diameter, 11-mm long, 0.25 pitch, and 0.2 NA) attached at distal end. The interference signal is detected by the on-board dual-balanced detectors and sampled by the 12 bit, 500  MS/s FPGA DAQ board in the OCT engine. Depth-resolved OCT B-scans are generated in real time by the onboard FPGA processor and streamed to the experimental control computer for visualization. The OCT system has an axial resolution of 10.2  μm and a transverse resolution of 16.3  μm in the focal plane.

### 2.8 Multivariate Analysis of FLIm Parameters

Pixel-wise Pearson correlation coefficient between all fluorescence lifetime images (channels 1 to 4) and intensity ratio images (channels 1 to 4) was analyzed using MATLAB (MathWorks, Natick, Massachusetts, USA). Image data were filtered by a threshold signal-to-noise ratio (SNR) value of 25 in channel 3 (channel with the lowest signal intensity) to remove data points with low SNR. The same filter was applied to data from all channels to ensure that the dimension data are identical. The correlation coefficient was average across all images irrespective of treatment or imaging time point. Correlation matrix is visualized using R statistical software (Foundation for Statistical Computing, Vienna) and p<0.05 were considered statistically significant.

### 2.9 Biochemical Homogeneous Index

Statistical homogeneity theory^36^ was used to determine tissue homogeneity using FLIm lifetime data of channel. FLIm channel 1 lifetime was chosen based on the result of multivariant analysis of FLIm parameters. To compute the homogeneity index, a ring-shaped region of interest centered on the centroid of the tissue image was first selected so that the area of the ROI was half of the total area of the sample. The mean fluorescence lifetime of the ROI was computed. The percentage of the FLIm pixels inside the ROI that falls between (1±0.05) × mean lifetime was defined as the homogeneity index. If all the pixels inside the ROI fall within (1±0.05) × mean lifetime, the homogeneity index would be 1.

### 2.10 Histology

All samples were harvested following FLIm-OCT assessment at 4 weeks *in vivo* and fixed in 10% neutral buffered formalin, paraffin embedded, sectioned at 10  μm, and stained with hematoxylin and eosin (H&E) for general morphology. The sections were imaged using an inverted microscope (BZ-X700, Keyence).

## 3 Results

### 3.1 FLIm Detects Heterogeneous Construct Growth

**Fig. 2:**
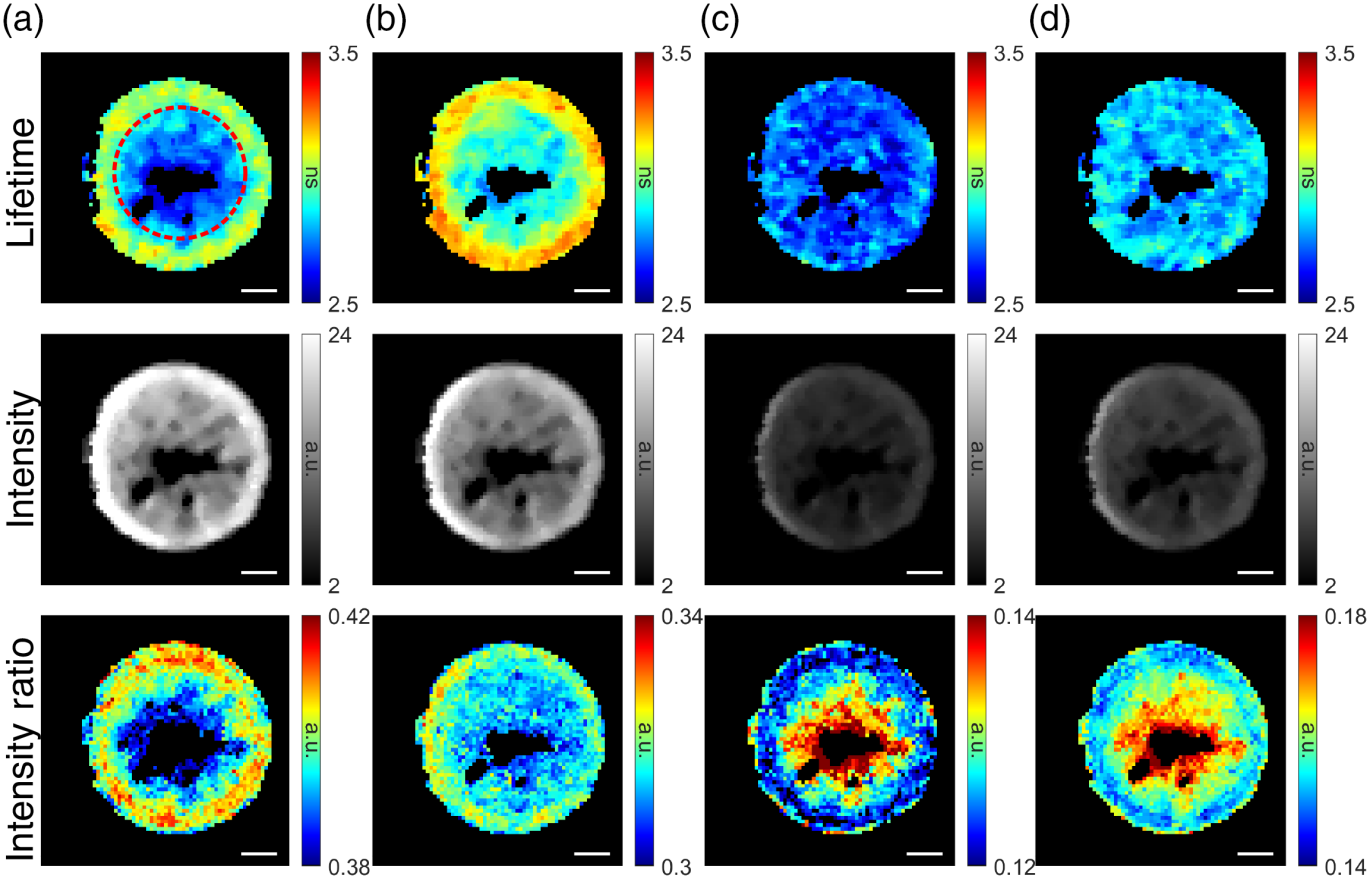
Representative FLIm maps of engineered tissue sample from LAP group at t=4 weeks during *in vitro* culture. Heterogenous growth of tissue construct was observed. The outer edge of the tissue sample exhibited longer lifetime and higher intensity ratio in FLIm channels (a) 1 and (b) 2. Relative constant lifetime values were observed across the whole construct in channels (c) 3 and (d) 4, with lower intensity ratio at the outer edge. Scale bar=1  mm.

Nondestructive FLIm assessment of engineered tissue construct during *in vitro* culture revealed heterogeneous tissue growth in all constructs at t=2 weeks and t=4 weeks for both treatment groups. Representative FLIm images of tissue construct at t=4 weeks are shown in Fig. 2. The outer edge of the tissue construct exhibited longer lifetime (Ch1: 3.1171±0.2371  ns; Ch2: ∼3.3606±0.2593  ns) and intensity ratio than the center of the construct (Ch1: 2.8861±0.1290  ns; Ch2: 3.1878±0.0970  ns) in both channels 1 and 2. Relative homogeneous lifetime was observed in channel 3 (2.7060±0.1162  ns) and 4 (2.8977±0.1201  ns) with lower intensity ratio on the outer edge of the tissue construct. A defect (void) was observed in the center of the tissue construct.

### 3.2 Multivariant Analysis of FLIm Data

**Fig. 3:**
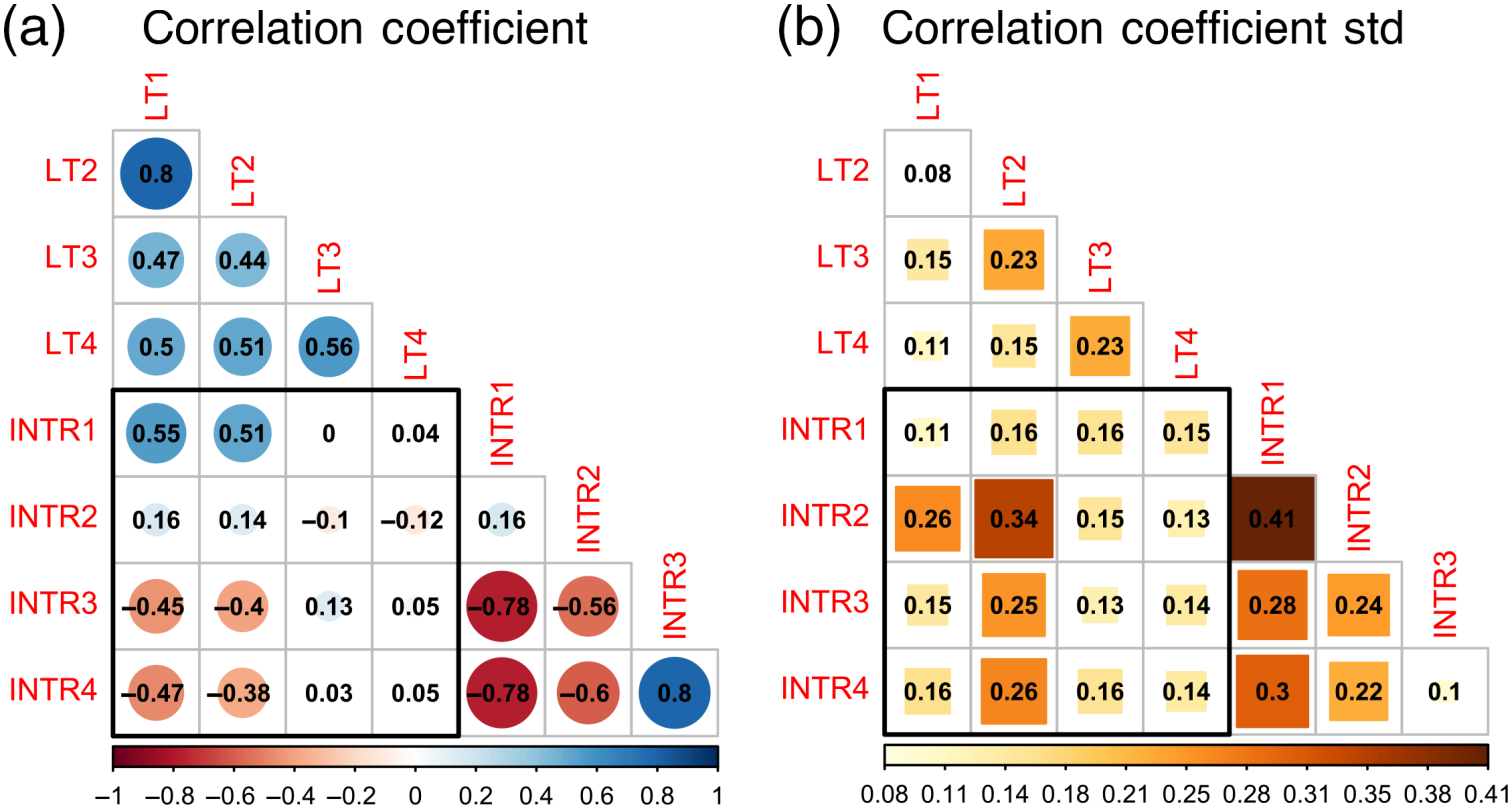
Correlogram of FLIm lifetimes and intensity ratios of channels 1 to 4. (a) The value of the correlation coefficients between variables pairs displayed and visualized in color. Red indicates negative correlation while blue indicates positive correlation. The size of the circle reflects the absolute value of the correlation coefficient. (b) The values of the correlation coefficient standard deviation displayed and visualized in color. The size of the square indicates the absolute value of the standard deviation. LT, lifetime; INTR, intensity ratio. Coefficient between lifetimes and intensity ratios are highlighted by the black square.

Average correlation matrix of all FLIm maps, irrespective of treatments or time points, are visualized in Fig. 3(a). The standard deviation of the correlation matrix is visualized in Fig. 3(b). All fluorescence lifetimes (LT1, LT2, LT3, and LT4) were correlated with each other. Among them, the highest correlation coefficient (0.8) existed between channel 1 lifetime (LT1) and channel 2 lifetime (LT2) with the lowest standard deviation of 0.08. Among intensity ratios, strongest correlation (0.8) was observed between channel 3 intensity ratio (INTR3) and channel 4 intensity ratio (INTR4) with the lowest standard deviation of 0.1. Channel 1 intensity ratio (INTR1) was inversely correlated with the intensity ratio of channel 3 (INTR3) and channel 4 (INTR4) with identical coefficient but positively with channel 1 lifetime (LT1) and channel 2 lifetime (LT2). Between lifetime and intensity ratios, channel 3 and channel 4 lifetimes had a weak or no correlation between all intensity ratios. Channel 1 intensity ratio (INTR1) was strongly correlated to channel 1 lifetime (LT1) and channel 2 lifetime (LT2).

### 3.3 Longitudinal FLIm Assessment of Construct Maturation

**Fig. 4:**
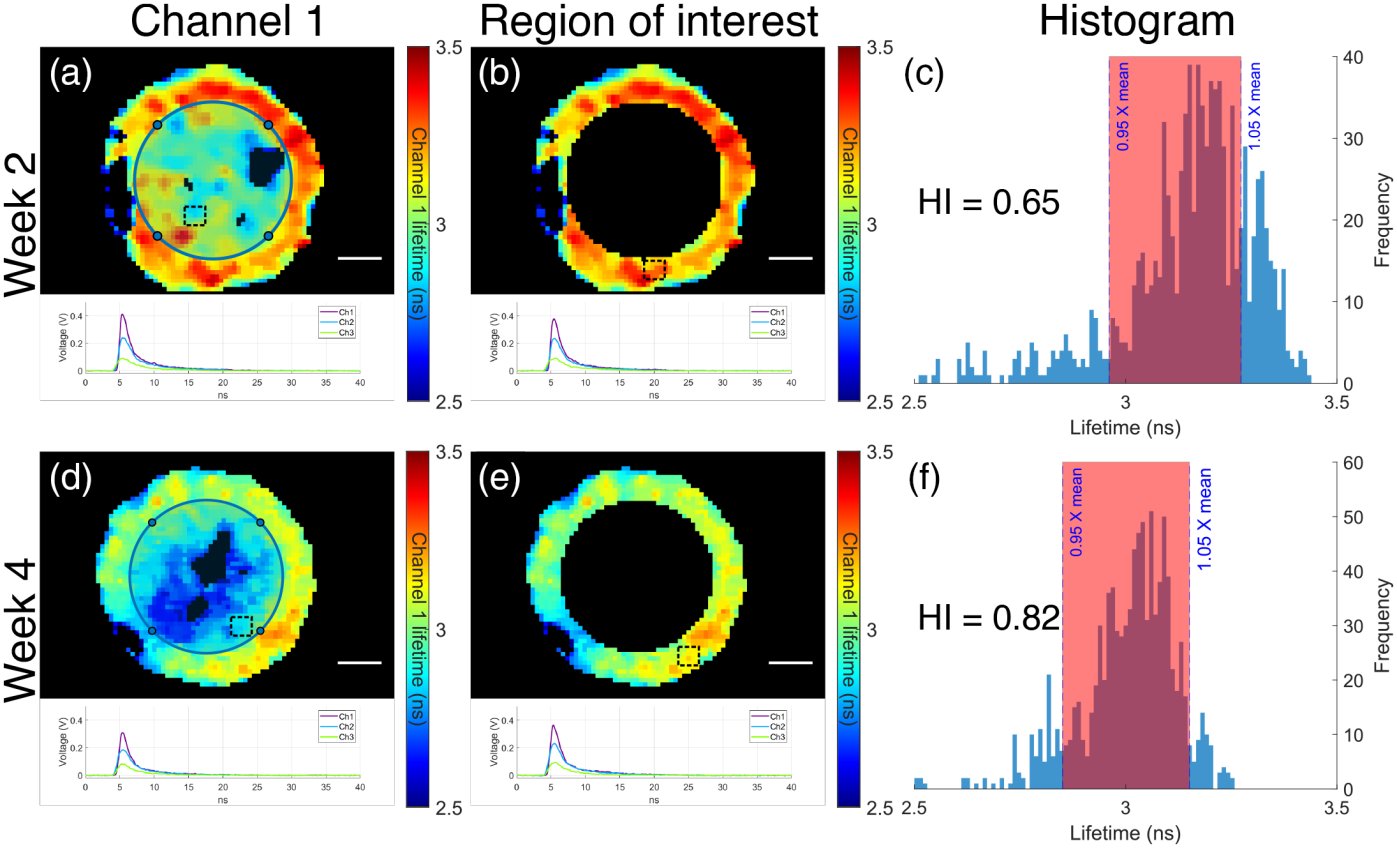
Longitudinal FLIm assessment detected tissue construct maturation. (a), (d) Representative channel 1 FLIm images of CTL group at (a) week 2 and (d) week 4. Circle at the center of the sample had an area that was half the area of the total sample. (b), (e) Regions of interest used for calculation of homogeneous index. Fluorescence waveforms averaged over the region highlighted by the black square were plotted. (c), (f) Histograms of channel 1 lifetime within the region of interest at (c) week 2 and (f) week 4. Scale bar=1  mm.

**Fig. 5:**
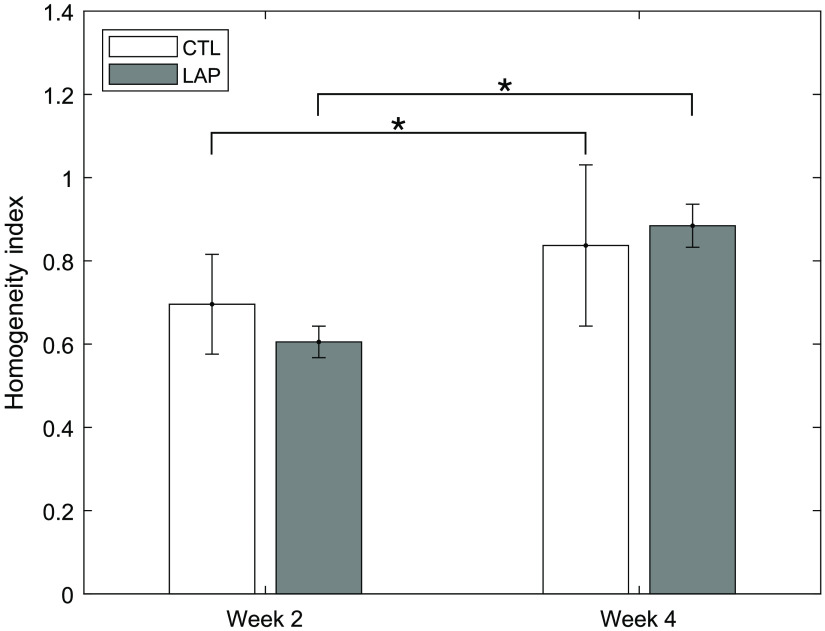
Homogeneity index (HI) of tissue construct. Significant increase (p<0.05) of HI was observed between week 2 and week 4 for both CTL and LAP group. No significant difference was observed between groups at either time points. *p<0.05.

Longitudinal FLIm assessment detected changes of tissue construct homogeneity index during *in vitro* culture. FLIm images of sample 2 from CTL group at t=2 weeks and t=4 weeks of *in vitro* culture are shown in Figs. 4(a) and 4(d), respectively. The ROIs used for homogeneity index calculation are shown in Figs. 4(b) and 4(e). The histogram of the fluorescence lifetime inside the ROI is plotted in Figs. 4(c) and 4(d), respectively. The homogeneity index of the tissue sample was higher for t=4 weeks as more (82%) of the pixels in the ROI fell between ±10% of the mean lifetime of ROI, compared to that of week 2 (62%). A bar plot of the homogeneity index (HI) at t=2 weeks and t=4 weeks for CTL and LAP group is shown in Fig. 5. Significant increase of HI was observed between week 2 and week 4 for both CTL and LAP group. No significant difference was observed between groups at either time point.

### 3.4 OCT Detected Structural Defect in Tissue Construct

**Fig. 6:**
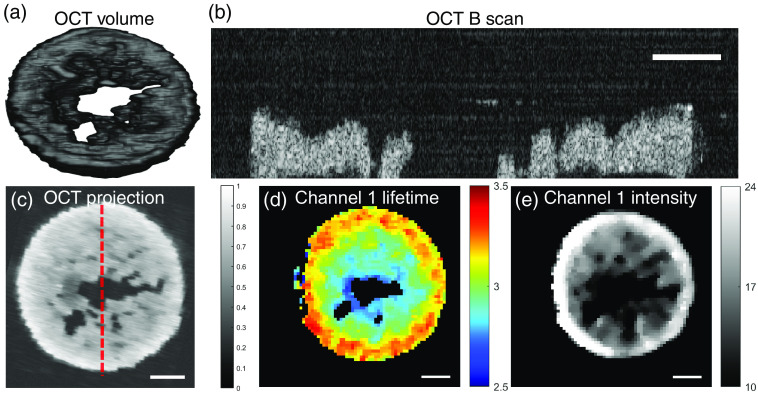
Nondestructive OCT assessment detected defect (void) in tissue construct. Matched sample with Fig. 2: (a) 3D volume rendering of tissue construct; (b) OCT B-scan from the center of the tissue construct, location indicated by red line in OCT projection; (c) enface projection of the 3D volumetric OCT data; (d) channel 1 fluorescence lifetime map of the sample; and (e) channel 1 fluorescence intensity map of the tissue sample. Scale bar=1  mm.

OCT assessment of the tissue construct during *in vitro* culture discovered defects (voids) at the center of tissue construct at both t=2 weeks and t=4 weeks, in agreement with FLIm assessment. A representative 3D volume rendering of the tissue construct (same sample as Fig. 2) is visualized in Fig. 6(a). A B-scan image from the center of the construct is shown in Fig. 6(b), and the location of the B-scan is indicated by red line in the enface projection of the OCT 3D volume data shown in Fig. 6(c). Tissue morphology measured by OCT matched well with that from fluorescence lifetime [Fig. 6(d)] and intensity [Fig. 6(e)] map.

### 3.5 Visualization of Construct in Vivo by FLIm-OCT

**Fig. 7:**
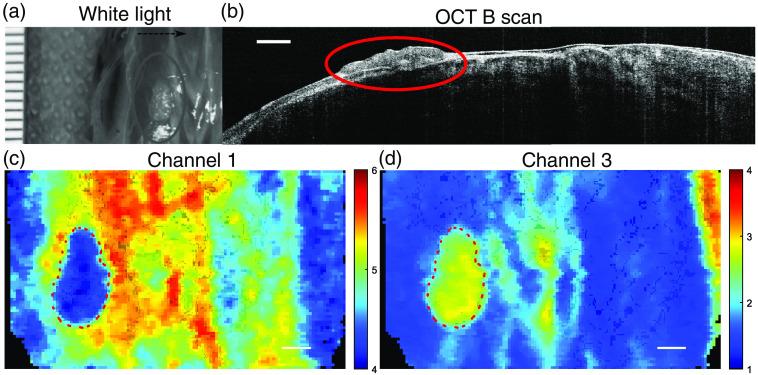
*In situ* FLIm and OCT image of cartilage construct on the back of nude mouse at t=4 weeks of *in vivo* culture. (a) White light image of the construct. Black arrow indicates scanning fast axis direction; (b) OCT B-scan image from the center of the construct; (c) FLIm channel 1 intensity weighted lifetime map. Construct ROI highlighted in red; and (d) FLIm channel 3 intensity weighted lifetime map. Construct ROI highlighted in red. Scale bar=1  mm.

Representative *in situ* FLIm and OCT images of tissue construct at t=4 weeks of *in vivo* culture are shown in Fig. 7. The tissue construct retained its hyaline color as shown in the white light image [Fig. 7(a)], whereas the size of the construct was reduced compared to t=4 weeks *in vitro* (Fig. 2). Tissue construct was visible in OCT B-scan image with distinct morphology from the muscle tissue of the mouse, which had a layered structure and smooth surface. The tissue construct exhibited lower lifetime (4.36±0.22  ns) in channel 1 compared to the surrounding native tissue (∼5 to 6 ns). The opposite was observed for channel 3 with tissue construct exhibited longer lifetime (2.58±0.24  ns) compared to the native tissue (∼1 to 2.5 ns).

### 3.6 OCT Detects Cyst Formation in Tissue Constructs

**Fig. 8:**
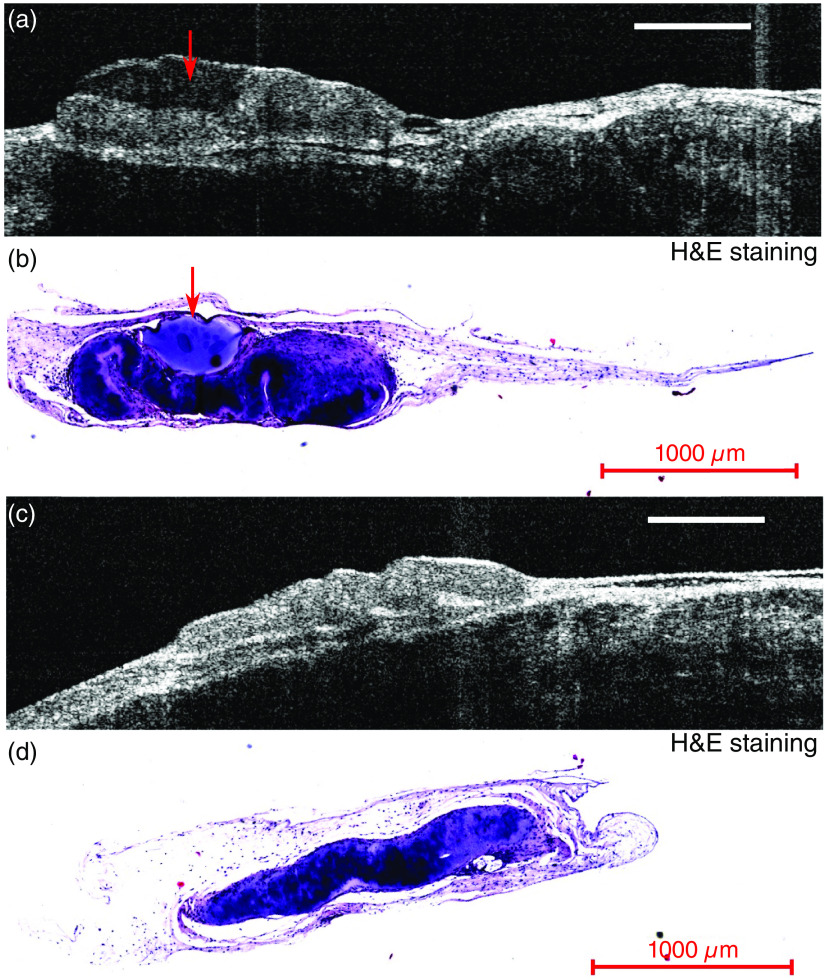
OCT image versus histology: (a) OCT B-scan images of a CTL tissue construct with a cyst (red arrow, hypoechoic region) forming in its center; (b) histology (H&E) of the tissue construct in (a) confirming the existence of cyst; (c) OCT B-scan image of a LAP tissue construct without cyst; and (d) histology (H&E) of the tissue construct shown in (c). Scale bar=1  mm.

Comparison of *in situ* OCT B scan image with histology (H&E) showed the formation of a cyst inside a CTL tissue construct that can be visualized by OCT. The cyst can be seen in the OCT B-scan image as a hypoechoic region with low intensity [Fig. 8(a)]. Histology (H&E) of the same tissue construct, as shown in Fig. 8(b), confirmed the presence of the cyst. For comparison, the OCT B-scan image and histology from an LAP tissue construct without the presence of cysts are shown in Figs. 7(c) and 7(d).

## 4 Discussion

This study is the first attempt, to the best of our knowledge, to monitor the maturation of engineered cartilage using multimodal FLIm-OCT during both *in vitro* culture and *in vivo* preclinical subcutaneous animal model. The results of this study demonstrated the applicability of a nondestructive imaging system, combining FLIm and OCT, for spatial and temporal monitoring of the biochemical and structural characteristics of the tissue engineered cartilage. This multimodal imaging system holds great potential as a quality control method to provide full characterization of tissue engineered neocartilage before its release for clinical use, thus reducing the expense of biomanufacturing significantly and facilitating the translation of engineered cartilage product into clinical use.

The self-assembling process is an approach that allows engineering of articular cartilage constructs without the use of exogenous scaffolds.^31^ Successful growth of self-assembled cartilage was associated by significant increase in construct collagen and GAGs content, thereby providing a target for optical detection. TGF-β has become one of the most used mediators for cartilage tissue engineering^37^^–^^39^ due to its ability to promote chondrogenesis and strongly improve the synthesis of an array of cartilaginous structural matrix proteins, including proteoglycans and collagen (type-II).^40^^–^^43^ TGF-β is often exogenously supplemented in culture media in its active form under the assumption that it will diffuse into constructs uniformly and improve the biosynthesis throughout the tissue. However, recent studies have shown that at the later stages of tissue development, due to the presence of nonspecific binding sites in the ECM of articular cartilage, active TGF-β accumulates exclusively in the construct periphery leading to highly heterogeneous construct growth.^32^ In this study, the heterogeneous tissue growth due to nonuniform distribution of TGF-β was observed *in vitro* in both groups at both imaging time points. The outer edge of the tissue construct exhibited high fluorescence lifetime and intensity ratio in channel 1 (375 to 410 nm) and channel 2 (450 to 485 nm) as shown in Fig. 2, indicating deposition of collagen content as a result of construct growth. The fluorescence lifetimes in channel 3 (CH3=532 to 565 nm) were relatively homogenous, which is not surprising as channel 3 is outside the collagen emission spectrum and mostly sensitive to fluorescence signals from cells. This result demonstrated the potential of FLIm as a tool for nondestructive monitoring of construct homogeneity during culture, which is critical for creating clinically relevant-sized constructs for joint repair.

Pixel-wise multivariant analysis of the FLIm data revealed strong correlation between fluorescence lifetime from all channels as shown in Fig. 3, with the strongest correlation between channels 1 and 2 lifetimes. This is expected as the major biological fluorophores in cartilage tissue construct, namely collagens, GAGs, and chondrocytes all have broad emission spectrum. Collagen has a high quantum yield and fluoresces predominantly in channels 1 and 2. GAGs fluoresce mostly in channel 3 with very low quantum yield, whereas cell fluorescence is present in channels 1 to 3.^15^ The high correlation between channels 1 and 2 can be explained by the high quantum yield of collagen, which will cause collagen fluorescence to dominate the signal collected in channel 1 and 2 giving rise to high correlation coefficient. The relative homogenous distribution of channel 3 lifetime and the lack of correlation between channel 3 lifetime with intensity ratios indicated that fluorescence from a single fluorophore was detected in channel 3. For engineered cartilage, this fluorophore is likely FAD inside chondrocytes. FAD has a broad emission from 500 to 600 nm matching the collection spectrum of channel 3.^44^ In addition, channel 3 fluorescence lifetime value of 2.70 ns agrees with the lifetime of FAD reported in the literature. Channel 4 lifetime exhibited similar trends as channel 3 but the origin of channel 4 fluorescence signal is currently unclear to us and needs further investigation. Based on this result, we identified the parameters that are most useful for identifying the change of construct biochemical components: FLIm channel 1 parameters can provide information about collagen content in construct ECM, whereas FLIm channel 3 parameters can be used to infer metabolic states of chondrocytes in the tissue constructs. On the other hand, we do acknowledge that FLIm is an imaging modality with limited penetration depth (∼300  μm), which will provide biochemical information limited to tissue construct periphery. In this study, FLIm is well suited to characterize the growth of engineered tissue constructs as the constructs are relatively thin and transparent. For other applications, the limited penetration depth of FLIm must be taken into consideration when interpreting imaging data.

The development of a biochemical homogeneity index based on *en face* FLIm parameters provided a single value to compare sample biochemical homogeneity among groups and across timepoints. The biochemical homogeneity index provides a method for quantifying the heterogenous growth of the construct, currently missing in destructive testing methods. Significant increase of the homogeneity index was observed between t=2 weeks and t=4 weeks, indicating tissue maturation. A ring-shaped ROI was chosen for this study to account for the circular symmetry of tissue sample but to exclude the defect (void) at the center of the construct. The size of the ROI was set to half of the total area of the sample to make sure the analysis was objective and consistent across all samples. The defect at the center of the construct is a structural feature and can be quantified more accurately using 3D OCT volume data therefore, OCT was excluded from the biochemical homogeneity index calculation. We acknowledge that our current definition of biochemical homogeneity index is sensitive to experimental noise (a completely homogenous sample with low fluorescent yield might show a homogeneity index <1 due to the significant variation in the lifetime at each pixel caused by noise); however, this can be minimized by filtering the FLIm data using SNR such that pixels with high noise are excluded from the analysis. In addition, a more robust biochemical homogeneity index can be developed to account for the experimental noise but is out of the scope of this paper.

A structural homogeneity index based on OCT data such as void volume, total sample volume, sample thickness or a combination of above parameters could provide a useful adjunct to the FLIm data to provide a more comprehensive evaluation of engineered tissue maturation. Such an analysis is not reported in this study due to unforeseen challenges caused by imaging artifact presented in the OCT data. In this study, in order to maintain the sterile tissue culture environment, tissue-engineered cartilage samples were imaged inside their culturing media, the surface of which reflects OCT light strongly and results in significant imaging artifacts in some of the OCT images making robust quantitative analysis of OCT data very challenging. This challenge can be easily mitigated by submerging the imaging probe inside the culturing media for future studies or applications.

Defects (voids) with varying size, which were not identifiable by naked eye due to the transparent color of cartilage construct, were detected by FLIm and OCT independently in all tissue construct *in vitro*, as shown in Figs. 2 and 6, respectively. The morphology of the tissue construct visualized by FLIm and OCT was very similar, as shown in Figs. 6(c)–6(e). However, the spatial distribution of collagen in ECM shown in FLIm channel 1 was not visible in OCT data. This result demonstrates that the complementary nature of FLIm and OCT techniques and the combination of both can yield a rapid and more complete characterization of tissue construct. The reason for the defect is unknown at this moment; more studies are needed to identify the reason for its formation.

FLIm-OCT assessment of the tissue construct in the preclinical animal model demonstrates that FLIm-OCT can be used to visualize the morphology and structure of the tissue construct *in situ* as shown in Fig. 7. The formation of a cyst in tissue construct was detected by OCT and confirmed with histology (Fig. 8). The size different of the tissue construct between OCT and histology can be contributed to the error in histology sectioning and tissue shrinking during histology processing. The advantage of *in situ* OCT imaging is that it can provide real-time cross-section image with resolution close to histology at any location nondestructively at multiple time points. Histology analysis only allows the visualization of the tissue structure at discrete point and precludes the possibility to evaluate the entire clinical sample prior to implantation due to its destructive nature. Only 5 out of 10 implanted samples (2 from CTL, 3 from LAP) survived the *in vivo* culture, the remaining were degraded by the mechanical forces present *in vivo*. As a result of the small sample size, quantitative analysis could not be performed on the *in vivo* imaging data.

Compared to ultrasound (US), which has been used previously for characterization of engineered tissue constructs,^19^ OCT provides higher resolution image with faster speed at the expense of penetration depth. For engineered cartilage, which is small in size and highly transparent, OCT is preferable compared to US. In this study, OCT is able to image the full depth of the constructs at every stage of maturation. But we acknowledge that for larger tissue samples, US could be a better modality than OCT due to its higher penetration depth at the expense of resolution. In addition, FLIm and OCT are both optical modalities, making the optical integration easy. A double clad fiber probe with diameter <400  μm can be easily utilized to deliver the 355 nm excitation of FLIm and 1310 nm lighter of OCT to tissue sample as demonstrated previously.^35^

## 5 Conclusion

This study demonstrates that multimodal FLIm-OCT technique can be used to nondestructively monitor the heterogenous growth of engineered tissue construct during its maturation prior to implantation. The combination of FLIm and OCT offers a method for spatial and temporal monitoring of the maturation of engineered tissue construct by probing *in situ* its chemical composition and structural properties. It is also flexible and can be easily extended to environment such as bioreactors or incubators. This approach has a profound benefit in clinical research and large-scale manufacturing of tissue engineered medical product where conventional quality control methods are destructive, sacrificing a large amount of tissue construct and incurring a large additional cost. Imaging parameters can be used to identify the optical/best sample to implant that will lead to best clinical outcome as well as tracking tissue maturation after implantation. Successful adoption of this method would accelerate the clinical translation of tissue engineered medical product from benchtop to bedside.

## Code, Data, and Materials availability

Data underlying the results presented in this paper may be obtained from the authors upon reasonable request and through a collaboration agreement.

## References

[1] Hunziker E. B., “Articular cartilage repair: are the intrinsic biological constraints undermining this process insuperable?” Osteoarthr. Cartilage 7, 15–28 (1999). OSCAEO 1063-4584 10.1053/joca.1998.0159 10367012

[2] Steadman J. R. Rodkey W. G. Rodrigo J. J., “Microfracture: surgical technique and rehabilitation to treat chondral defects,” Clin. Orthop. Relat. Res. 391, S362–S369 (2001). CORTBR 0009-921X 10.1097/00003086-200110001-00033 11603719

[3] Jackson D. W. Simon T. M. Aberman H. M., “Symptomatic articular cartilage degeneration: the impact in the new millennium,” Clin. Orthop. Relat. Res. 391 Suppl, S14–S25 (2001). CORTBR 0009-921X 10.1097/00003086-200110001-00003 11603698

[4] Makris E. A. et al., “Repair and tissue engineering techniques for articular cartilage,” Nat. Rev. Rheumatol. 11, 21–34 (2015). 10.1038/nrrheum.2014.157 25247412 PMC4629810

[5] Castro N. J. et al., “Nondestructive testing of native and tissue-engineered medical products: adding numbers to pictures,” Trends Biotechnol. 40(2), 194–209 (2021). TRBIDM 0167-7799 10.1016/j.tibtech.2021.06.009 34315621 PMC8772387

[6] Harrison R. P. Medcalf N. Rafiq Q. A., “Cell therapy-processing economics: small-scale microfactories as a stepping stone toward large-scale macrofactories,” Regen. Med. 13, 159–173 (2018). 10.2217/rme-2017-0103 29509065

[7] Haudenschild A. K. et al., “Nondestructive fluorescence lifetime imaging and time-resolved fluorescence spectroscopy detect cartilage matrix depletion and correlate with mechanical properties,” Eur. Cell Mater. 36, 30–43 (2018). 10.22203/eCM.v036a03 30051455

[8] Moisio K. et al., “Denuded subchondral bone and knee pain in persons with knee osteoarthritis,” Arthritis Rheum. 60, 3703–3710 (2009). 10.1002/art.25014 19950284 PMC2833327

[9] Bian L. et al., “Influence of decreasing nutrient path length on the development of engineered cartilage,” Osteoarthr. Cartilage 17, 677–685 (2009). OSCAEO 1063-4584 10.1016/j.joca.2008.10.003 PMC3387279 19022685

[r10] Hung C. T. et al., “Anatomically shaped osteochondral constructs for articular cartilage repair,” J. Biomech. 36, 1853–1864 (2003). JBMCB5 0021-9290 10.1016/S0021-9290(03)00213-6 14614939

[11] Hung C. I. et al., “A paradigm for functional tissue engineering of articular cartilage via applied physiologic deformational loading,” Ann. Biomed. Eng. 32, 510–510 (2004). ABMECF 0090-6964 10.1023/B:ABME.0000017816.70785.d8 14964720

[12] Gorpas D. et al., “Real-time visualization of tissue surface biochemical features derived from fluorescence lifetime measurements,” IEEE Trans. Med. Imaging 35, 1802–1811 (2016). ITMID4 0278-0062 10.1109/TMI.2016.2530621 26890641 PMC5131727

[13] Berezin M. Y. Achilefu S., “Fluorescence lifetime measurements and biological imaging,” Chem. Rev. 110, 2641–2684 (2010). CHREAY 0009-2665 10.1021/cr900343z 20356094 PMC2924670

[14] Georgakoudi I. et al., “NAD(P)H and collagen as in vivo quantitative fluorescent biomarkers of epithelial precancerous changes,” Cancer Res. 62, 682–687 (2002). CNREA8 0008-5472 11830520

[15] Alfonso-Garcia A. et al., “Fiber-based fluorescence lifetime imaging of recellularization processes on vascular tissue constructs,” J. Biophotonics 11, e201700391 (2018). 10.1002/jbio.201700391 29781171 PMC7700018

[16] Harvestine J. N. et al., “Multimodal label-free imaging for detecting maturation of engineered osteogenic grafts,” ACS Biomater. Sci. Eng. 5, 1956–1966 (2019). 10.1021/acsbiomaterials.9b00007 33405522 PMC8594456

[17] Mitra D. et al., “Detection of pentosidine cross-links in cell-secreted decellularized matrices using time resolved fluorescence spectroscopy,” ACS Biomater. Sci. Eng. 3, 1944–1954 (2017). 10.1021/acsbiomaterials.6b00029 28944287 PMC5604893

[18] Haudenschild A. K. et al., “Non-destructive detection of matrix stabilization correlates with enhanced mechanical properties of self-assembled articular cartilage,” J. Tissue Eng. Regen. Med. 13, 637–648 (2019). 10.1002/term.2824 30770656 PMC6461514

[19] Sun Y. et al., “Nondestructive evaluation of tissue engineered articular cartilage using time-resolved fluorescence spectroscopy and ultrasound backscatter microscopy,” Tissue Eng. Part C Methods 18, 215–226 (2012). 10.1089/ten.tec.2011.0343 22010819 PMC3285603

[20] Huang D. et al., “Optical coherence tomography,” Science 254, 1178–1181 (1991). SCIEAS 0036-8075 10.1126/science.1957169 1957169 PMC4638169

[21] Brezinski M. E. Fujimoto J. G., “Optical coherence tomography: high-resolution imaging in nontransparent tissue,” IEEE J. Sel. Top. Quantum 5, 1185–1192 (1999). IJSQEN 1077-260X 10.1109/2944.796345

[22] Yabushita H. et al., “Characterization of human atherosclerosis by optical coherence tomography,” Circulation 106, 1640–1645 (2002). CIRCAZ 0009-7322 10.1161/01.CIR.0000029927.92825.F6 12270856

[23] Wessels R. et al., “Optical biopsy of epithelial cancers by optical coherence tomography (OCT),” Laser Med. Sci. 29, 1297–1305 (2014). 10.1007/s10103-013-1291-8 PMC4031426 23504262

[24] Jahr H. Brill N. Nebelung S., “Detecting early stage osteoarthritis by optical coherence tomography?” Biomarkers 20, 590–596 (2015). 10.3109/1354750X.2015.1130190 26862954 PMC4819848

[25] El-Haddad M. T. Tao Y. K., “Advances in intraoperative optical coherence tomography for surgical guidance,” Curr. Opin. Biomed. Eng. 3, 37–48 (2017). 10.1016/j.cobme.2017.09.007

[26] Kim K. Wagner W. R., “Non-invasive and non-destructive characterization of tissue engineered constructs using ultrasound imaging technologies: a review,” Ann. Biomed. Eng. 44, 621–635 (2016). ABMECF 0090-6964 10.1007/s10439-015-1495-0 26518412 PMC4792694

[27] Fite B. Z. et al., “Noninvasive multimodal evaluation of bioengineered cartilage constructs combining time-resolved fluorescence and ultrasound imaging,” Tissue Eng. Part C Methods 17, 495–504 (2011). 10.1089/ten.tec.2010.0368 21303258 PMC3065732

[28] Yang Y. et al., “Investigation of optical coherence tomography as an imaging modality in tissue engineering,” Phys. Med. Biol. 51, 1649–1659 (2006). PHMBA7 0031-9155 10.1088/0031-9155/51/7/001 16552095

[29] Sherlock B. E. et al., “Simultaneous, label-free, multispectral fluorescence lifetime imaging and optical coherence tomography using a double-clad fiber,” Opt. Lett. 42, 3753–3756 (2017). OPLEDP 0146-9592 10.1364/OL.42.003753 28957119 PMC8951707

[30] Chu C. R. Szczodry M. Bruno S., “Animal models for cartilage regeneration and repair,” Tissue Eng. Part B Rev. 16, 105–115 (2010). 10.1089/ten.teb.2009.0452 19831641 PMC3121784

[31] Hu J. C. Athanasiou K. A., “A self-assembling process in articular cartilage tissue engineering,” Tissue Eng. 12, 969–979 (2006).1937-3341 10.1089/ten.2006.12.969 16674308

[32] Albro M. B. et al., “Heterogeneous engineered cartilage growth results from gradients of media-supplemented active TGF-beta and is ameliorated by the alternative supplementation of latent TGF-beta,” Biomaterials 77, 173–185 (2016). BIMADU 0142-9612 10.1016/j.biomaterials.2015.10.018 26599624 PMC4968414

[33] Yankelevich D. R. et al., “Design and evaluation of a device for fast multispectral time-resolved fluorescence spectroscopy and imaging,” Rev. Sci. Instrum. 85, 034303 (2014). RSINAK 0034-6748 10.1063/1.4869037 24689603 PMC3971822

[34] Liu J. et al., “A novel method for fast and robust estimation of fluorescence decay dynamics using constrained least-squares deconvolution with Laguerre expansion,” Phys. Med. Biol. 57, 843–865 (2012). PHMBA7 0031-9155 10.1088/0031-9155/57/4/843 22290334 PMC3407553

[35] Sherlock B. E. et al., “Synchronous fluorescence lifetime imaging and optical coherence tomography using a double clad fiber,” in IEEE Photonics Conf., pp. 1–2 (2016). 10.1109/IPCon.2016.7831145

[36] Luo Z. P., “Statistical quantification of the microstructural homogeneity of size and orientation distributions,” J. Mater. Sci. 45, 3228–3241 (2010). JMTSAS 0022-2461 10.1007/s10853-010-4330-x

[37] Byers B. A. et al., “Transient exposure to transforming growth factor beta 3 under serum-free conditions enhances the biomechanical and biochemical maturation of tissue-engineered cartilage,” Tissue Eng. Part A 14, 1821–1834 (2008).1937-3341 10.1089/ten.tea.2007.0222 18611145 PMC2656914

[r38] Huang A. H. et al., “Transient exposure to transforming growth factor beta 3 improves the mechanical properties of mesenchymal stem cell-laden cartilage constructs in a density-dependent manner,” Tissue Eng. Part A 15, 3461–3472 (2009).1937-3341 10.1089/ten.tea.2009.0198 19432533 PMC2792068

[39] Revell C. M. Reynolds C. E. Athanasiou K. A., “Effects of initial cell seeding in self assembly of articular cartilage,” Ann. Biomed. Eng. 36, 1441–1448 (2008). ABMECF 0090-6964 10.1007/s10439-008-9524-x 18574692 PMC3164522

[40] Galera P. et al., “Transforming growth factor-beta-1 (TGF-β-1) up-regulation of collagen type-II in primary cultures of rabbit articular chondrocytes (RAC) involves increased messenger-RNA levels without affecting messenger-RNA stability and procollagen processing,” J. Cell Physiol. 153, 596–606 (1992). 10.1002/jcp.1041530322 1447320

[r41] Hiraki Y. et al., “Effect of transforming growth factor beta on cell proliferation and glycosaminoglycan synthesis by rabbit growth-plate chondrocytes in culture,” Biochim. Biophys. Acta 969, 91–99 (1988). BBACAQ 0006-3002 10.1016/0167-4889(88)90092-4 3162385

[r42] Morales T. I. Roberts A. B., “Transforming growth factor beta regulates the metabolism of proteoglycans in bovine cartilage organ cultures,” J. Biol. Chem. 263, 12828–12831 (1988). JBCHA3 0021-9258 10.1016/S0021-9258(18)37634-8 3166454

[43] Redini F. et al., “Transforming growth factor beta stimulates collagen and glycosaminoglycan biosynthesis in cultured rabbit articular chondrocytes,” Febs. Lett. 234, 172–175 (1988). FEBLAL 0014-5793 10.1016/0014-5793(88)81327-9 3164687

[44] Marcu L. French P. M. Elson D. S., Fluorescence Lifetime Spectroscopy and Imaging: Principles and Applications in Biomedical Diagnostics, CRC Press (2014).

